# Mobile phone usage duration and male fertility: A two-sample Mendelian randomization analysis

**DOI:** 10.1097/MD.0000000000044668

**Published:** 2025-09-19

**Authors:** Yang Xiang, Lan Xu, Yuechen Sun, Chaomin Hu, Leyao Lv

**Affiliations:** a Department of Emergency, Huzhou Central Hospital, Affiliated Huzhou Hospital Zhejiang University School of Medicine, Huzhou, Zhejiang, China.

**Keywords:** male fertility, Mendelian randomization, mobile phone use

## Abstract

Mobile phone use has become ubiquitous in modern life, raising public concern over its potential effects on male reproductive health. While several observational studies have reported associations between prolonged phone use and decreased sperm quality or testosterone levels, these findings remain inconclusive due to residual confounding, reverse causation, and inconsistent exposure measurement. We conducted a two-sample Mendelian randomization (MR) analysis using genetic variants associated with mobile phone usage duration to investigate its causal effects on male fertility. Inverse variance weighting (IVW) was employed as the primary analytical method. Genetic predisposition to longer mobile phone use was not associated with levels of sex hormone-binding globulin (SHBG), total testosterone, or the risk of abnormal sperm parameters, erectile dysfunction, or testicular dysfunction. These null findings were consistent across all MR methods and sensitivity analyses. Our findings suggest that mobile phone use is unlikely to have a direct causal impact on male reproductive hormones or sexual function. Future research incorporating precise exposure measurements and mechanistic evaluations is warranted.

## 1. Introduction

Male infertility refers to the situation in which men are unable to promote the pregnancy of their female partners under normal reproductive conditions. Male infertility contributes to approximately 50% of infertility cases worldwide^[1]^ and is influenced by a range of factors, including genetic predisposition, hormonal imbalances, lifestyle, and environmental exposures.^[2]^ Recent evidence indicates a consistent decline in global sperm concentration, estimated at 1.4% to 1.6% per year.^[3]^ This trend has prompted increasing scrutiny of potential environmental contributors, including electromagnetic radiation emitted by mobile phones. Several observational studies have suggested an association between mobile phone use and impaired semen quality, particularly abnormal sperm parameters.^[4]^ However, these findings remain inconclusive due to methodological limitations such as residual confounding, reverse causation, and exposure misclassification.^[5,6]^ Consequently, the causal nature of this association remains uncertain.

Evidence from observational studies remains susceptible to confounding and reverse causation that cannot be fully excluded in observational studies. Thus, the direction of causality between length of phone use and male fertility is unclear. Mendelian randomization (MR) is an epidemiological approach that infers exposure-outcome causality from observational data used by genetic variants as instrumental variables (IVs), which overcomes these limitations. Based on Mendel laws that each allele is randomly transmitted to the offspring with an equal probability, the risk of confounding and reverse causation is greatly reduced.^[7,8]^ Besides, the unidirectional flow of biological information from the parental generation to progeny can limit the effects of reverse causation.^[9]^ In this study, we applied a two-sample MR design to assess whether mobile phone usage duration has a causal impact on male fertility outcomes, including levels of sex hormones (SHBG and total testosterone) and sexual dysfunction (abnormal sperm, erectile dysfunction, and testicular dysfunction).

## 2. Methods

### 2.1. Study design

Genetic variants associated with mobile phone usage duration were selected as IVs to investigate their potential causal effects on male fertility indicators, including sex hormones (SHBG and total testosterone) and sexual dysfunction (abnormal sperm, erectile dysfunction, and testicular dysfunction) in population-based studies.

**Figure 1.**
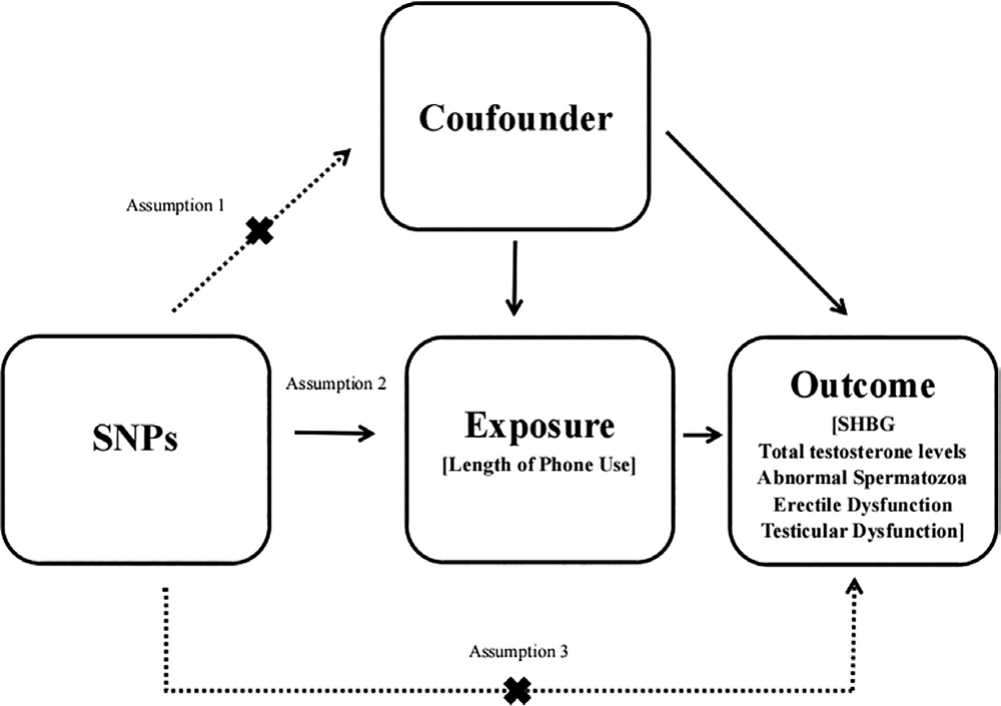
Schematic diagram illustrating the study design.

MR validity relies on 3 core assumptions: relevance – IVs must be strongly associated with the exposure; independence – IVs must not be associated with confounders; and exclusion restriction – IVs influence the outcome only through the exposure of interest (Fig. 1).^[8]^

### 2.2. Data sources

Summary-level genetic association data for mobile phone usage duration were obtained from the UK Biobank, comprising 501,296 individuals. Genome-wide association studies (GWAS) summary statistics for sex hormone-binding globulin (SHBG) (IEU-B-4870), total testosterone (ebi-a-GCST90012114), and abnormal sperm (finn-b-R18_ABNORMAL_SPERMATOZ) were retrieved from the IEU OpenGWAS database (https://gwas.mrcieu.ac.uk/).

The SHBG dataset included 214,989 participants and 12,321,875 single-nucleotide polymorphisms (SNPs); the total testosterone dataset comprised 425,097 individuals and 16,321,861 SNPs. For abnormal sperm, 209,926 cases, 209,006 controls and 16,380,442 SNPs were analyzed. Data for erectile dysfunction (2418 cases, 179,346 controls) and testicular dysfunction (106 cases, 176,319 controls) were obtained from the FinnGen consortium. All participants were of European ancestry.

All data were obtained from publicly available GWAS summary statistics, and no additional ethical approval or participant consent was required.^[10,11]^

### 2.3. Genetic instrumental variable selection

Independent SNPs were selected as IVs based on a genome-wide significance threshold (*P* < 5 × 10^−8^) and linkage disequilibrium (*r*^2^ < 0.01, window = 1 Mb).^[12]^ A detailed list of the selected SNPs and their *F*-statistics is provided in Tables S1A to S1E (Supplemental Digital Content, https://links.lww.com/MD/Q69). To reduce pleiotropic bias, we excluded SNPs linked to known confounders or outcomes using the IEU OpenGWAS platform (https://gwas.mrcieu.ac.uk/). Palindromic SNPs with ambiguous strand orientation were removed.^[13]^ Instrument strength was evaluated using *F*-statistics (*F* > 10) and *R*^2^ to avoid weak instrument bias.^[14]^

### 2.4. Mendelian randomization analysis

The primary MR analysis employed inverse variance weighting (IVW).^[15]^ Sensitivity analyses included MR-Egger regression, weighted median (WM), and MR pleiotropy residual sum and outlier (MR-PRESSO) to account for horizontal pleiotropy and assess robustness. When there are invalid IVs in the genetic variation under study (such as pleiotropy of IVs), we need to use the WM method instead of the inverse variance weighted method for causality estimation. This method combines multiple genetic variants to estimate causal effects, allowing up to half of the invalid instrument variables to exist, and is a supplement to MR Egger method.^[16]^ The MR Egger method allows all IVs to be invalidated though at the expense of lower precision in causal estimation.^[17]^ To account for multiple testing across the 5 outcomes, we applied a Bonferroni correction, adjusting the significance threshold to α = 0.01 (0.05/5).

The MR-Egger intercept test was used to detect directional pleiotropy (*P* < .05).^[18,19]^ Cochran *Q* statistic and *I*^2^ were used to evaluate heterogeneity.^[18]^ MR-PRESSO was applied to identify and correct for outlier SNPs.^[18,20]^ Leave-one-out analysis was conducted to test whether causal estimates were driven by individual variants.^[21]^

All MR analyses were performed using the TwoSampleMR (MRC Integrative Epidemiology Unit, University of Bristol, Bristol, UK) and MR-PRESSO packages (Verbanck et al, McGill University, Montréal, Québec, Canada) in R (version 4.4.1; R Core Team, R Foundation for Statistical Computing, Vienna, Austria). This study used only publicly available, de-identified GWAS summary statistics, and thus ethical approval and informed consent were not required.

## 3. Results

**Figure 2.**
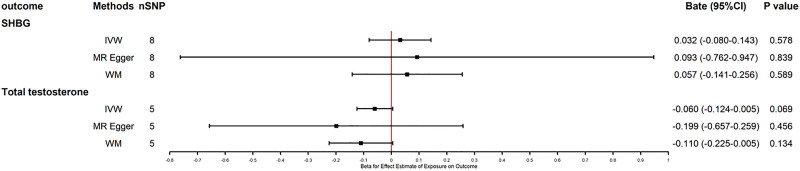
Forest plot showing MR estimates for SHBG and total testosterone. IVW = inverse variance weighted, SHBG = sex hormone-binding globulin, WM = weighted median.

**Figure 3.**
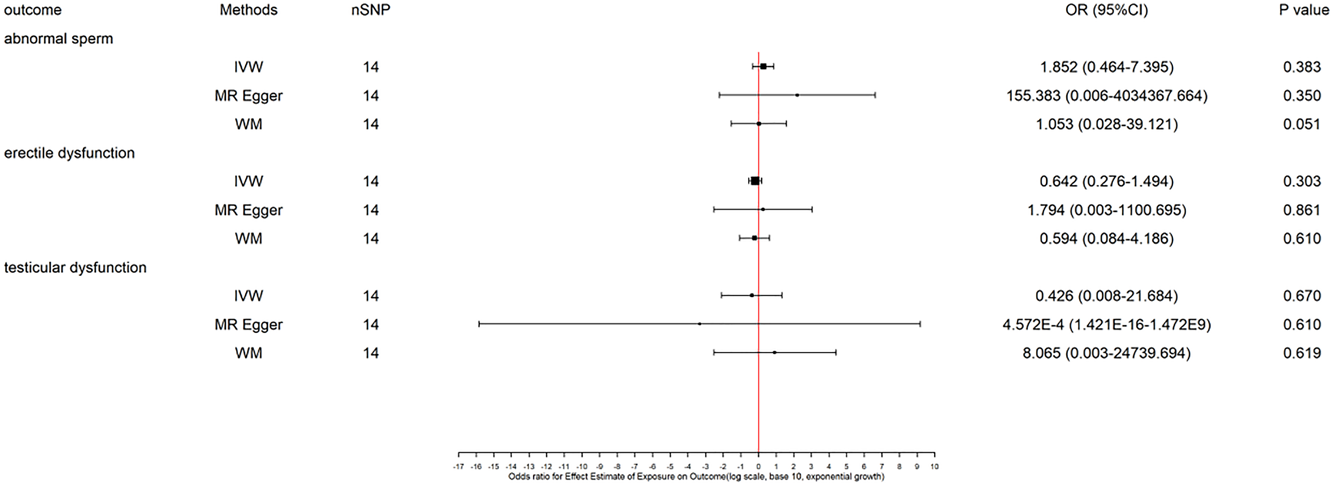
MR estimates for abnormal sperm, erectile dysfunction, and testicular dysfunction. IVW = inverse variance weighted, WM = weighted median.

The primary MR analyses using IVW showed no evidence of a causal effect of mobile phone usage duration on serum SHBG or total testosterone levels (Fig. 2). Similarly, no significant associations were observed between mobile phone use and abnormal sperm parameters, erectile dysfunction, or testicular dysfunction (Fig. 3).

These null findings remained consistent across all sensitivity analyses, including MR-Egger regression, WM, and MR-PRESSO. After Bonferroni correction, none of the associations remained statistically significant, further supporting the robustness of the null results. No evidence of directional pleiotropy or heterogeneity was detected (Table 1). Slight differences between IVW and MR-Egger estimates likely reflect the latter’s correction for potential directional pleiotropy. The nonsignificant MR-Egger intercepts further support the absence of substantial pleiotropy bias. Leave-one-out analyses and funnel plots were conducted to further assess the robustness of the MR estimates, and the results are presented in Supplementary Figures S1 to S5 (Supplemental Digital Content, https://links.lww.com/MD/Q69).

**Table 1:** Summary of Mendelian randomization estimates for the association between mobile phone usage duration and male fertility outcomes.

Outcome	MR method	Effect estimate LP on outcome			Test of pleiotropy	
		Beta/OR*	95% CI	*P*	Test	
SHBG	IVW	0.032	−0.080 to 0.143	.578	Cochran *Q* statistic (p)	1.571 (0.980)
	MR-Egger	0.093	−0.762 to 0.947	.839	MR-Egger intercept (p)	−0.001 (0.892)
	Weighted median	0.057	−0.141 to 0.256	.589	MR-PRESSO (p)	NA
Total testosterone	IVW	−0.060	−0.124 to 0.005	.069	Cochran *Q* statistic (p)	4.262 (0.372)
	MR-Egger	−0.199	−0.657 to 0.259	.456	MR-Egger intercept (p)	0.003 (0.588)
	Weighted median	−0.110	−0.225 to 0.005	.134	MR-PRESSO (p)	NA
Abnormal sperm	IVW	1.852	0.464 to 7.395	.383	Cochran *Q* statistic (p)	12.874 (0.458)
	MR-Egger	155.383	0.006 to 4,034,367.664	.350	MR-Egger intercept (p)	−0.091 (0.405)
	Weighted median	1.053	0.028 to 39.121	.051	MR-PRESSO (p)	NA
Erectile dysfunction	IVW	0.642	0.276 to 1.494	.303	Cochran *Q* statistic (p)	13.308 (0.424)
	MR-Egger	1.794	0.003 to 1100.695	.861	MR-Egger intercept (p)	−0.021 (0.757)
	Weighted median	0.594	0.084 to 4.186	.610	MR-PRESSO (p)	NA
Testicular dysfunction	IVW	0.426	0.008 to 21.68	.670	Cochran *Q* statistic (p)	4.717 (0.981)
	MR-Egger	0.457E−3	0.142E−15 to 10472E+9	.610	MR-Egger intercept (p)	0.139 (0.647)
	Weighted median	8.065	0.003 to 24,739.694	.619	MR-PRESSO (p)	NA

CI = confidence interval, IVW = inverse variance weighted, LP = length of phone use, NA = not available, OR = odds ratio, SHBG = sex hormone-binding globulin, SNPs = single-nucleotide polymorphisms.

* Estimates are presented as beta coefficients for continuous outcomes (SHBG, testosterone) and odds ratios for binary outcomes (abnormal sperm, erectile dysfunction, testicular dysfunction).

Taken together, these results indicate that genetically predicted mobile phone use does not causally affect male reproductive hormone levels or sexual function. The consistency across multiple MR approaches supports the robustness of our findings.

## 4. Discussion

In this two-sample MR study, we found no evidence that genetically predicted mobile phone usage duration has a causal effect on male fertility, as measured by SHBG and total testosterone levels, as well as indicators of sexual dysfunction including abnormal sperm, erectile dysfunction, and testicular dysfunction.

Several observational studies have reported associations between mobile phone use and impaired semen parameters, such as reduced sperm count or motility.^[4,22]^ However, findings remain inconsistent across studies, with some reports showing no significant effects.^[23]^ Potential mechanisms proposed in animal studies include oxidative stress, testicular apoptosis, and impaired sperm motility following radiofrequency electromagnetic field (RF-EMF) exposure.^[24–27]^ Another animal study concluded that the total sperm count of rats exposed to radiofrequency electromagnetic radiation from mobile phones did not change, but sperm motility decreased.^[28]^ South Korean academics discovered that cell phone radiation had no adverse effect on rat sperm.^[29]^ At present, there are no MR Studies on cell phone radiation and sperm status. Previous studies have shown that mobile phone use has a negative effect on erectile function.^[30]^ An animal experiment shows that long-term cell phone radiation can lead to testicular cell apoptosis in mice.^[31]^ Existing studies have not ruled out the potential effects of occupational, lifestyle, and other sources of electromagnetic waves on sexual function and sex hormones.

Notably, observational studies are inherently prone to confounding, reverse causality, and exposure misclassification.

The MR approach employed in our study mitigates these biases by leveraging genetic variants as proxies for mobile phone usage. Our consistent null findings across multiple MR methods – IVW, MR-Egger, WM, and MR-PRESSO – support a noncausal explanation for previously reported associations. Strengths of this study include the use of large-scale GWAS datasets, consistent MR findings across multiple sensitivity analyses, and restriction to participants of European ancestry, minimizing population stratification. Moreover, the robustness of our results across different methodological assumptions strengthens the validity of our conclusions. Given the multiple outcomes tested, the use of a conservative Bonferroni correction reduces the likelihood of false positive findings and supports the reliability of our null results. The slight variation between the IVW and MR-Egger estimates is expected, as the MR-Egger method adjusts for potential pleiotropy at the cost of reduced precision. The consistency of null findings across methods enhances confidence in the reliability of our causal interpretation.

Nonetheless, several limitations should be noted. First, our analysis was restricted to individuals of European ancestry and may not generalize to other populations. Second, mobile phone usage duration is self-reported, which may result in exposure misclassification and attenuated causal estimates despite the MR design mitigating confounding. Third, MR estimates reflect lifelong genetic predisposition and may not capture short-term or nonlinear effects of mobile phone use. Fourth, we could not account for variability in phone model, electromagnetic frequency, or usage patterns. Finally, due to limited GWAS data, we did not assess outcomes such as free androgen index or estradiol levels. Additionally, although the mean *F*-statistics for the selected instruments exceeded the conventional threshold, residual bias from weak instruments cannot be fully ruled out.

Moreover, MR relies on several key assumptions, including the absence of horizontal pleiotropy and the use of valid genetic proxies. Behavioral traits like mobile phone usage are particularly challenging to instrument genetically, which may compromise the accuracy of causal estimation.

## 5. Conclusion

In conclusion, this MR study provides no evidence for a causal relationship between mobile phone usage duration and male reproductive health, including sex hormone levels and sexual function. These findings suggest that previously reported associations may be attributable to confounding or reverse causation. Further research, including rigorously designed randomized trials and mechanistic studies, is warranted to further investigate the potential impact of radiofrequency electromagnetic exposure on male fertility. Our null findings suggest that previously observed associations between mobile phone use and male fertility may be attributable to residual confounding or reverse causation. Future studies should incorporate more precise exposure measurement, examine device-specific and frequency-specific effects, and explore biological mechanisms through experimental or longitudinal designs.

## Acknowledgments

We thank the UK Biobank, IEU OpenGWAS, and FinnGen consortia for providing access to publicly available summary-level GWAS data.

## Author contributions

**Conceptualization:** Yang Xiang.

**Data curation:** Yang Xiang, Lan Xu, Yuechen Sun, Chaomin Hu.

**Formal analysis:** Yang Xiang, Chaomin Hu.

**Writing – original draft:** Yang Xiang, Leyao Lv.

**Writing – review & editing:** Yang Xiang, Leyao Lv.

## Supplementary Material


