# Supplementary material for: Mobile phone usage duration and male fertility: A two-sample Mendelian randomization analysis
Source: Medicine (Baltimore). 2025 Sep 19;104(38):e44668. doi: 10.1097/MD.0000000000044668 (PMC12459597; doi:10.1097/MD.0000000000044668)

**Mobile Phone Usage Duration and Male Fertility: A Two-Sample Mendelian Randomization Analysis**

Yang Xiang1, Lan Xu1, Leyao Lv1*

**Supplementary Table S1A: List of SNPs used as instrumental variables for mobile phone usage duration in the analysis of sex hormone-binding globulin (SHBG), with effect alleles, beta coefficients, p-values, and F-statistics.**

| ****SNP**** | ****Effect Allele**** | ****Other Allele**** | ****Beta**** | ****P-value**** | ****F-statistic**** |
| --- | --- | --- | --- | --- | --- |
| rs10498394 | A | G | 0.0188045 | 0.780001 | 16.81556468 |
| rs11742919 | C | T | 0.0189938 | 0.36 | 10.90897217 |
| rs12035012 | C | A | -0.0273888 | 0.709999 | 17.74742865 |
| rs1397253 | G | A | 0.020002 | 0.760001 | 10.43136619 |
| rs17759770 | T | C | -0.0212381 | 0.74 | 12.98702833 |
| rs2760744 | T | C | -0.0193002 | 0.57 | 12.62469991 |
| rs2964252 | G | A | 0.0188151 | 0.68 | 13.80213424 |
| rs7860559 | T | C | 0.0180462 | 0.74 | 14.75077347 |

**Supplementary Table S1B: List of SNPs used as instrumental variables for mobile phone usage duration in the analysis of Total testosterone, with effect alleles, beta coefficients, p-values, and F-statistics.**

| ****SNP**** | ****Effect Allele**** | ****Other Allele**** | ****Beta**** | ****P-value**** | ****F-statistic**** |
| --- | --- | --- | --- | --- | --- |
| rs10207597 | C | T | 0.0178532 | 0.8 | 15.08671936 |
| rs1397253 | G | A | 0.020002 | 0.88 | 10.43136619 |
| rs34381905 | G | T | 0.0270734 | 0.0629999 | 11.63262078 |
| rs359265 | G | A | -0.0182596 | 0.15 | 15.2094801 |
| rs7860559 | T | C | 0.0180462 | 0.12 | 14.75077347 |

**Supplementary Table S1C: List of SNPs used as instrumental variables for mobile phone usage duration in the analysis of abnormal sperm, with effect alleles, beta coefficients, p-values, and F-statistics.**

| ****SNP**** | ****Effect Allele**** | ****Other Allele**** | ****Beta**** | ****P-value**** | ****F-statistic**** |
| --- | --- | --- | --- | --- | --- |
| rs10207597 | C | T | 0.0178532 | 0.8195 | 15.08671936 |
| rs10498394 | A | G | 0.0188045 | 0.4161 | 15.08671936 |
| rs11742919 | C | T | 0.0189938 | 0.0863595 | 16.81556468 |
| rs12035012 | C | A | -0.0273888 | 0.1942 | 10.90897217 |
| rs1397253 | G | A | 0.020002 | 0.9953 | 17.74742865 |
| rs17759770 | T | C | -0.0212381 | 0.3691 | 10.43136619 |
| rs2760744 | T | C | -0.0193002 | 0.0781592 | 12.98702833 |
| rs28397165 | T | C | -0.0210485 | 0.1758 | 12.62469991 |
| rs2964252 | G | A | 0.0188151 | 0.1972 | 11.10344345 |
| rs34381905 | G | T | 0.0270734 | 0.6145 | 13.80213424 |
| rs359265 | G | A | -0.0182596 | 0.7914 | 11.63262078 |
| rs4889891 | C | A | 0.0204787 | 0.673 | 15.2094801 |
| rs7860559 | T | C | 0.0180462 | 0.557799 | 13.78283453 |
| rs9650656 | A | G | 0.0175638 | 0.8378 | 13.20031961 |

**Supplementary Table S1D: List of SNPs used as instrumental variables for mobile phone usage duration in the analysis of erectile dysfunction, with effect alleles, beta coefficients, p-values, and F-statistics.**

| ****SNP**** | ****Effect Allele**** | ****Other Allele**** | ****Beta**** | ****P-value**** | ****F-statistic**** |
| --- | --- | --- | --- | --- | --- |
| rs10207597 | C | T | 0.0178532 | 0.927693 | 15.08671936 |
| rs10498394 | A | G | 0.0188045 | 0.714172 | 16.81556468 |
| rs11742919 | C | T | 0.0189938 | 0.0668529 | 10.90897217 |
| rs12035012 | C | A | -0.0273888 | 0.23767 | 17.74742865 |
| rs1397253 | G | A | 0.020002 | 0.911387 | 10.43136619 |
| rs17759770 | T | C | -0.0212381 | 0.606137 | 12.98702833 |
| rs2760744 | T | C | -0.0193002 | 0.921159 | 12.62469991 |
| rs28397165 | T | C | -0.0210485 | 0.148549 | 11.10344345 |
| rs2964252 | G | A | 0.0188151 | 0.173229 | 13.80213424 |
| rs34381905 | G | T | 0.0270734 | 0.897013 | 11.63262078 |
| rs359265 | G | A | -0.0182596 | 0.0828915 | 15.2094801 |
| rs4889891 | C | A | 0.0204787 | 0.444883 | 20.45991278 |
| rs7860559 | T | C | 0.0180462 | 0.336121 | 14.75077347 |
| rs9650656 | A | G | 0.0175638 | 0.391315 | 13.95537047 |

**Supplementary Table S1E: List of SNPs used as instrumental variables for mobile phone usage duration in the analysis of testicular dysfunction, with effect alleles, beta coefficients, p-values, and F-statistics.**

| ****SNP**** | ****Effect Allele**** | ****Other Allele**** | ****Beta**** | ****P-value**** | ****F-statistic**** |
| --- | --- | --- | --- | --- | --- |
| rs10207597 | C | T | 0.0178532 | 0.885738 | 15.08671936 |
| rs10498394 | A | G | 0.0188045 | 0.416153 | 16.81556468 |
| rs11742919 | C | T | 0.0189938 | 0.627138 | 10.90897217 |
| rs12035012 | C | A | -0.0273888 | 0.968644 | 17.74742865 |
| rs1397253 | G | A | 0.020002 | 0.748075 | 10.43136619 |
| rs17759770 | T | C | -0.0212381 | 0.47095 | 12.98702833 |
| rs2760744 | T | C | -0.0193002 | 0.409485 | 12.62469991 |
| rs28397165 | T | C | -0.0210485 | 0.66165 | 11.10344345 |
| rs2964252 | G | A | 0.0188151 | 0.353667 | 13.80213424 |
| rs34381905 | G | T | 0.0270734 | 0.369448 | 11.63262078 |
| rs359265 | G | A | -0.0182596 | 0.810747 | 15.2094801 |
| rs4889891 | C | A | 0.0204787 | 0.620299 | 20.45991278 |
| rs7860559 | T | C | 0.0180462 | 0.806912 | 14.75077347 |
| rs9650656 | A | G | 0.0175638 | 0.49956 | 13.95537047 |

**Supplementary Fig. 1A: Scatter plot showing the genetic associations with mobile phone usage duration (exposure) versus sex hormone-binding globulin (SHBG) levels (outcome). Each point represents an SNP. The lines show MR estimates using the inverse-variance weighted (IVW), MR-Egger, and weighted median methods.**


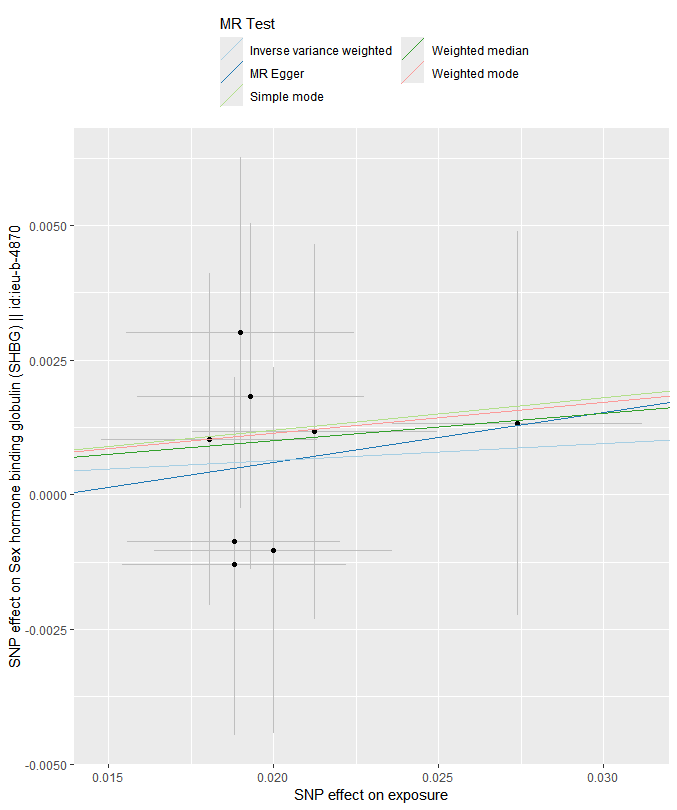


**Supplementary Fig. 1B: Leave-one-out sensitivity analysis for the association between mobile phone usage duration and SHBG. Each point represents the MR estimate obtained after omitting one SNP at a time. The stability of the estimates indicates that no single SNP has a disproportionate effect.**


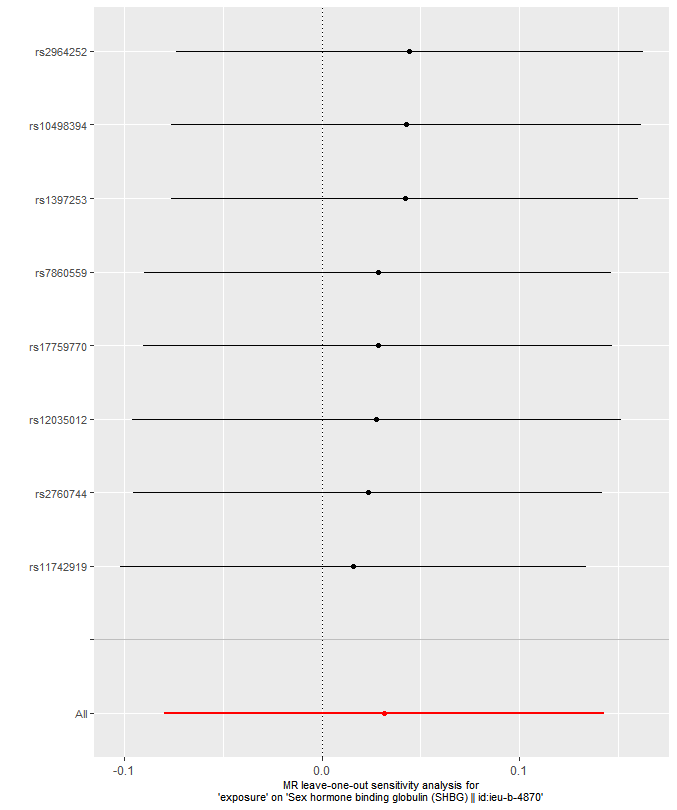


**Supplementary Fig. 1C: Forest plot showing the individual SNP estimates and the overall MR estimate for the association between mobile phone usage duration and SHBG using the IVW method.**


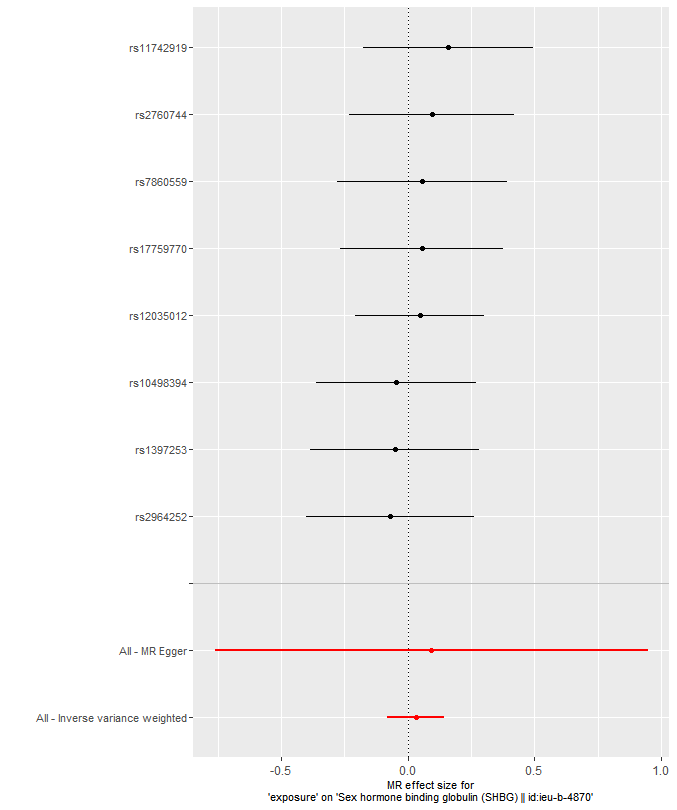


**Supplementary Fig. 1D: Funnel plot assessing potential small-study bias in the MR analysis of mobile phone usage duration and SHBG. The symmetry suggests no substantial directional pleiotropy.**


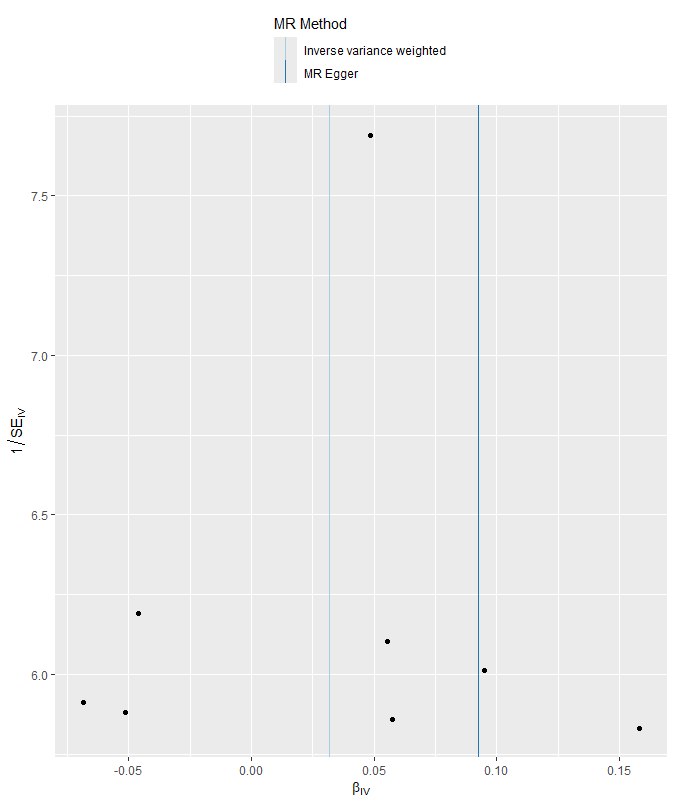


**Supplementary Fig. 2A: Scatter plot showing the genetic associations with mobile phone usage duration (exposure) versus total testosterone levels (outcome). Each point represents an SNP. The lines show MR estimates using the inverse-variance weighted (IVW), MR-Egger, and weighted median methods.**


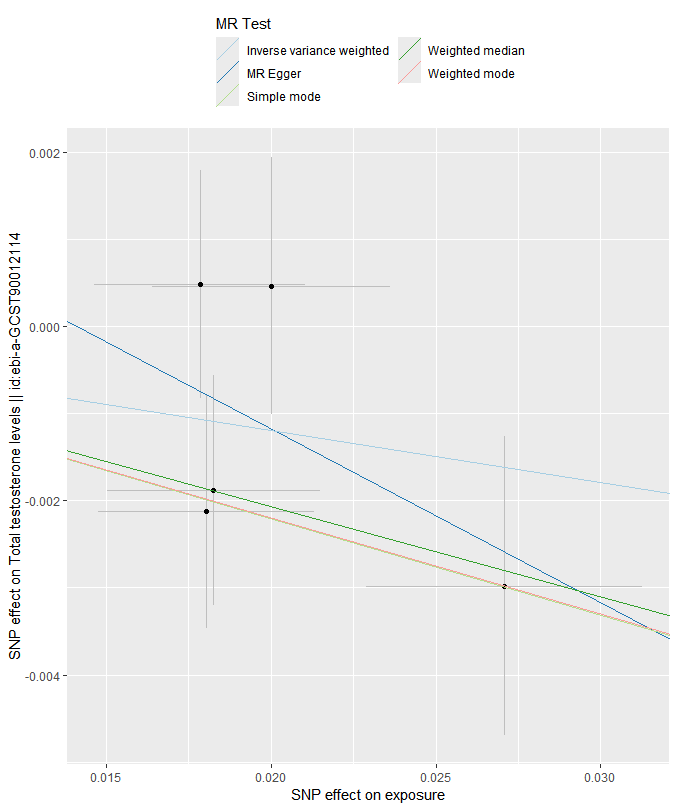


**Supplementary Fig. 2B: Leave-one-out sensitivity analysis for the association between mobile phone usage duration and total testosterone. Each point represents the MR estimate obtained after omitting one SNP at a time. The stability of the estimates indicates that no single SNP has a disproportionate effect.**


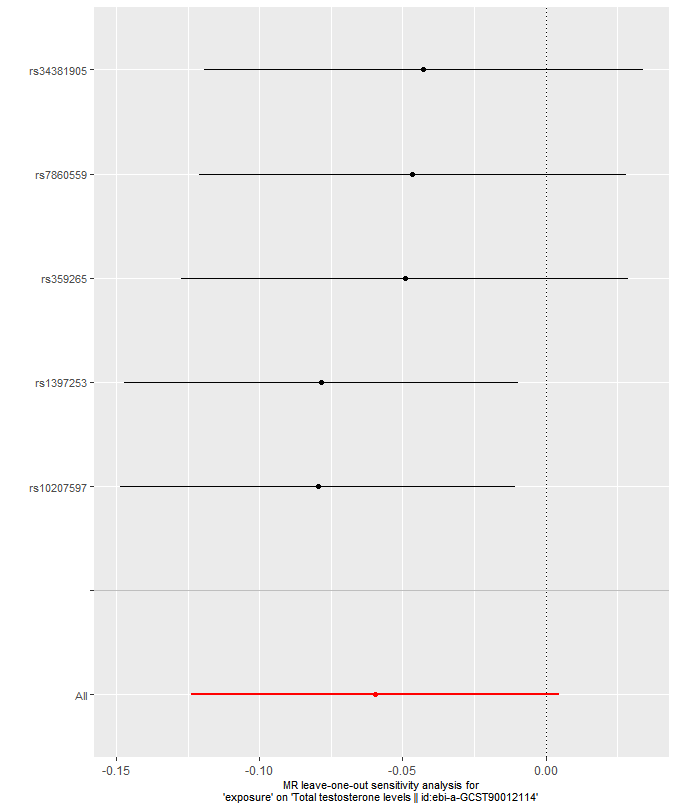


**Supplementary Fig. 2C: Forest plot showing the individual SNP estimates and the overall MR estimate for the association between mobile phone usage duration and total testosterone using the IVW method.**


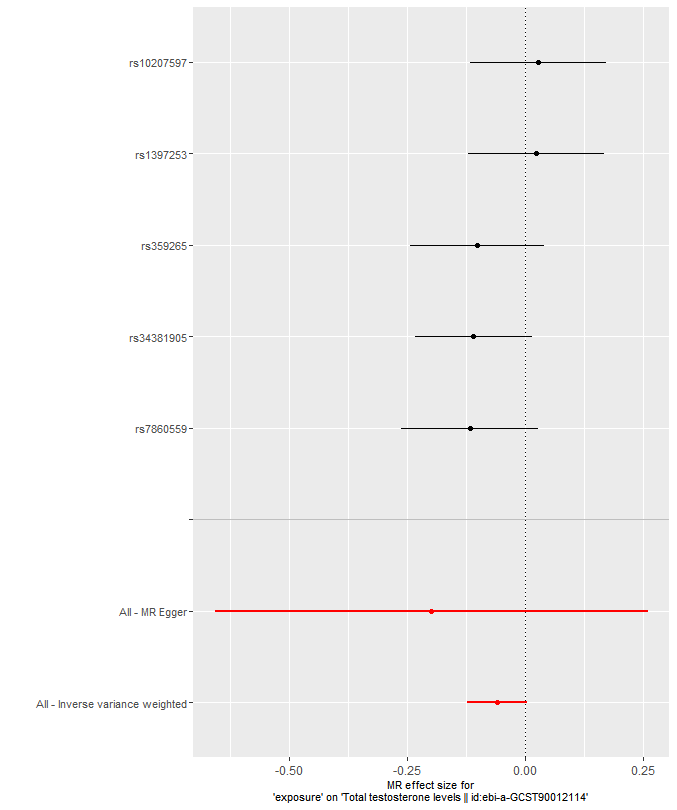


**Supplementary Fig. 2D: Funnel plot assessing potential small-study bias in the MR analysis of mobile phone usage duration and total testosterone. The symmetry suggests no substantial directional pleiotropy.**


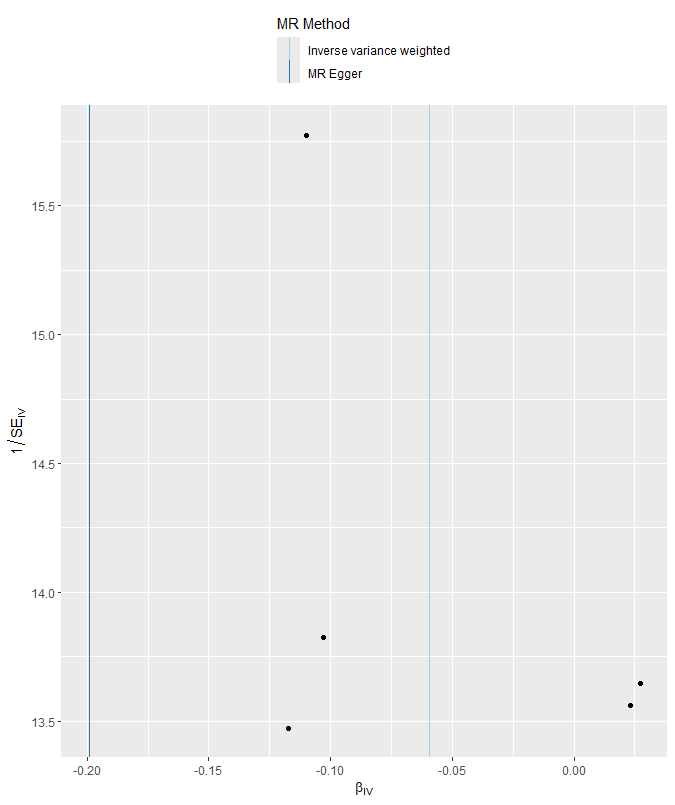


**Supplementary Fig. 3A: Scatter plot showing the genetic associations with mobile phone usage duration (exposure) versus abnormal sperm levels (outcome). Each point represents an SNP. The lines show MR estimates using the inverse-variance weighted (IVW), MR-Egger, and weighted median methods.**


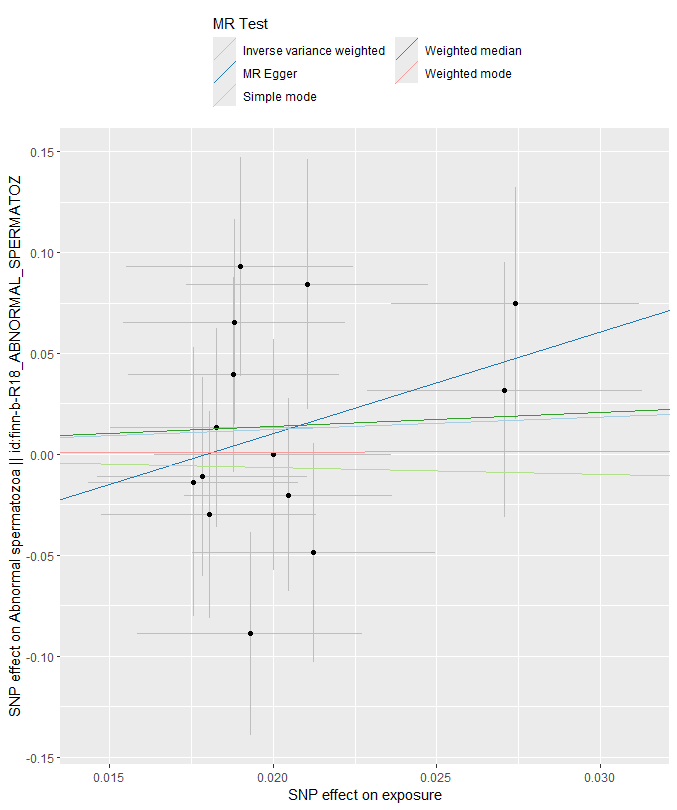


**Supplementary Fig. 3B: Leave-one-out sensitivity analysis for the association between mobile phone usage duration and abnormal sperm. Each point represents the MR estimate obtained after omitting one SNP at a time. The stability of the estimates indicates that no single SNP has a disproportionate effect.**


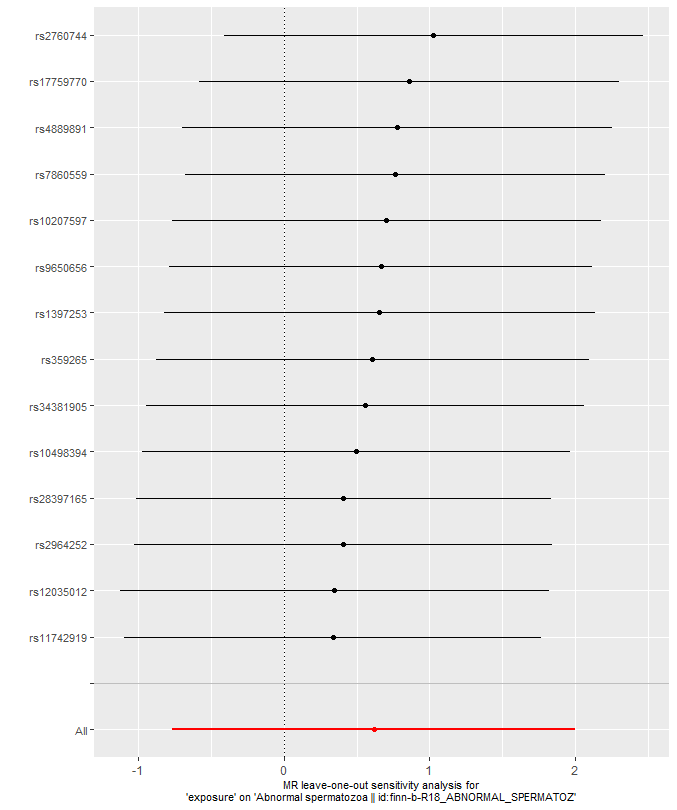


**Supplementary Fig. 3C: Forest plot showing the individual SNP estimates and the overall MR estimate for the association between mobile phone usage duration and abnormal sperm using the IVW method.**


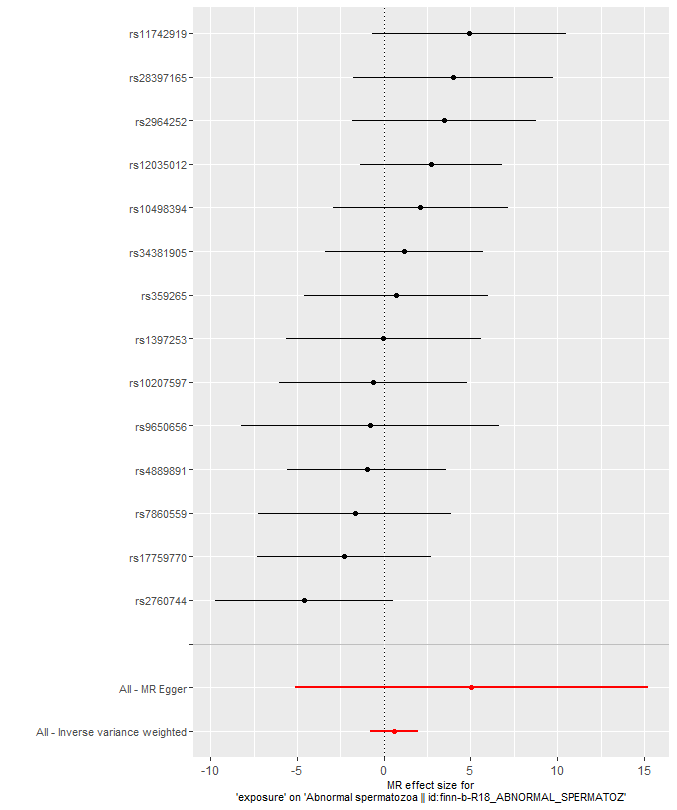


**Supplementary Fig. 3D: Funnel plot assessing potential small-study bias in the MR analysis of mobile phone usage duration and abnormal sperm. The symmetry suggests no substantial directional pleiotropy.**


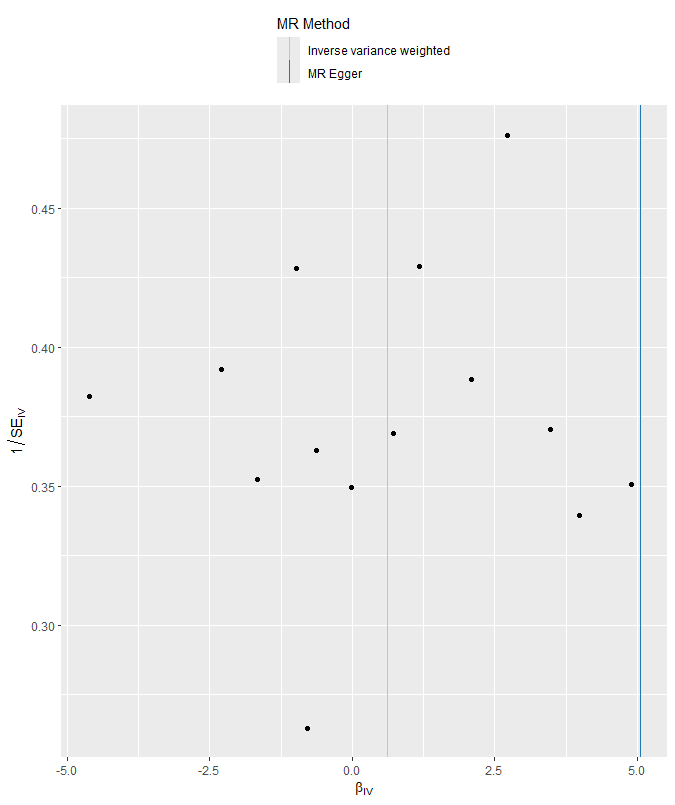


**Supplementary Fig. 4A: Scatter plot showing the genetic associations with mobile phone usage duration (exposure) versus erectile dysfunction levels (outcome). Each point represents an SNP. The lines show MR estimates using the inverse-variance weighted (IVW), MR-Egger, and weighted median methods.**


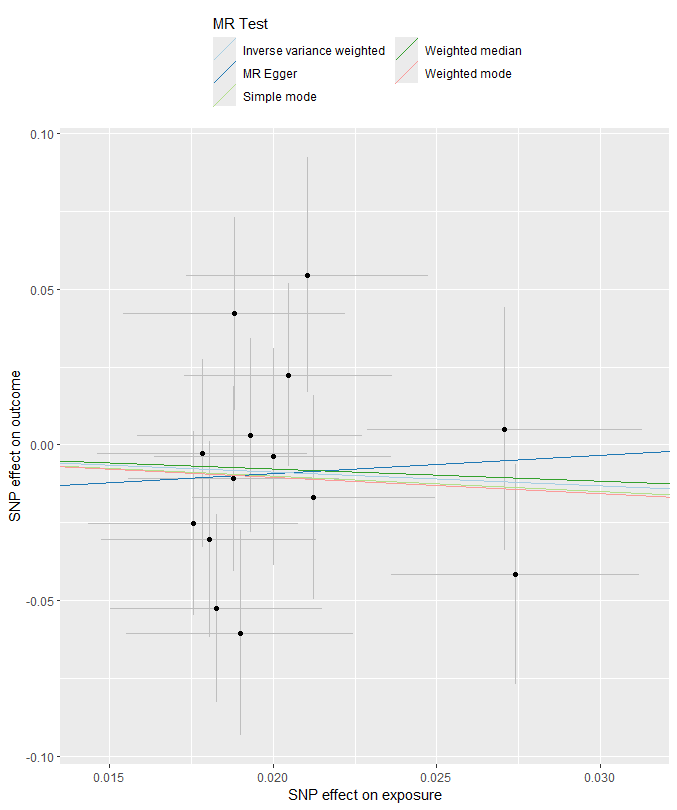


**Supplementary Fig. 4B: Leave-one-out sensitivity analysis for the association between mobile phone usage duration and erectile dysfunction. Each point represents the MR estimate obtained after omitting one SNP at a time. The stability of the estimates indicates that no single SNP has a disproportionate effect.**


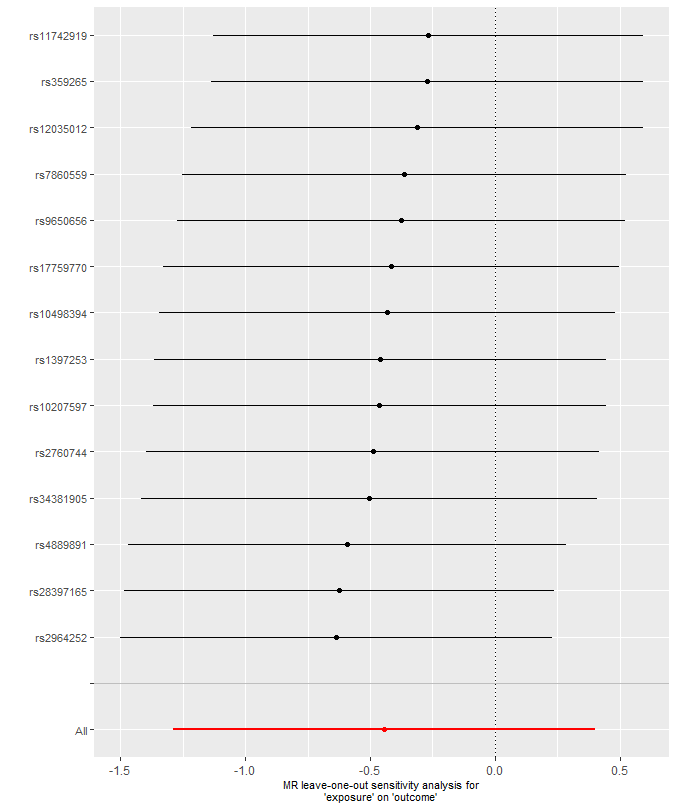


**Supplementary Fig. 4C: Forest plot showing the individual SNP estimates and the overall MR estimate for the association between mobile phone usage duration and erectile dysfunction using the IVW method.**


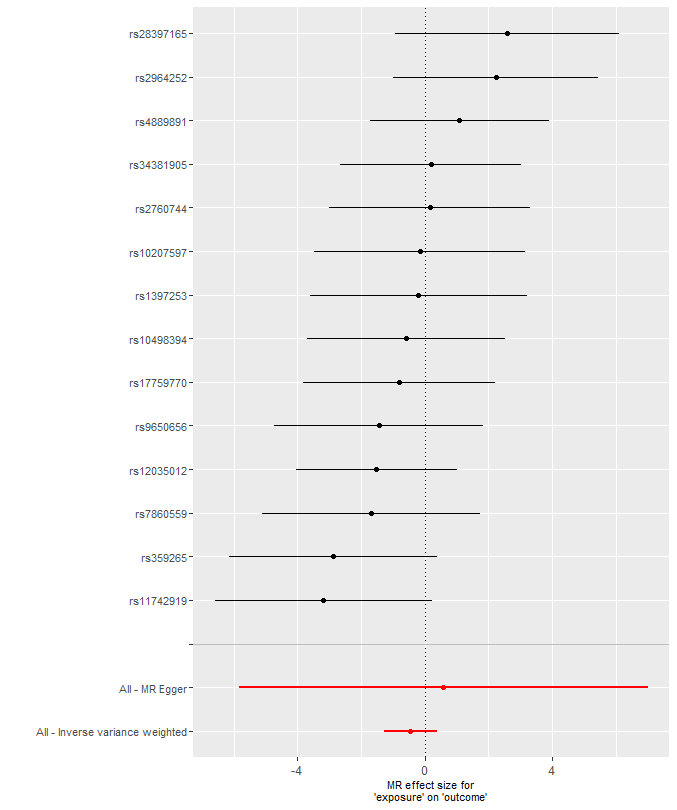


**Supplementary Fig. 4D: Funnel plot assessing potential small-study bias in the MR analysis of mobile phone usage duration and erectile dysfunction. The symmetry suggests no substantial directional pleiotropy.**


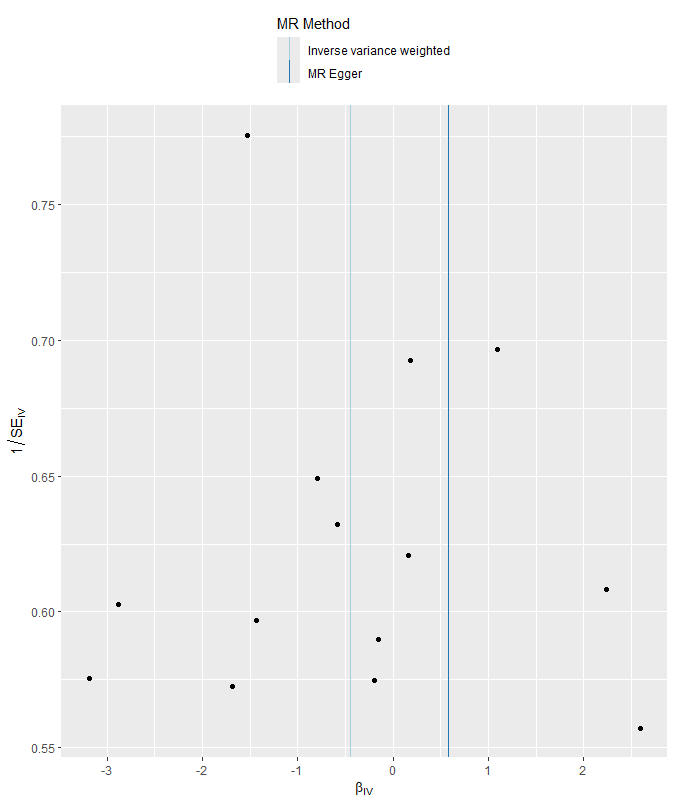


**Supplementary Fig. 5A: Scatter plot showing the genetic associations with mobile phone usage duration (exposure) versus testicular dysfunction levels (outcome). Each point represents an SNP. The lines show MR estimates using the inverse-variance weighted (IVW), MR-Egger, and weighted median methods.**


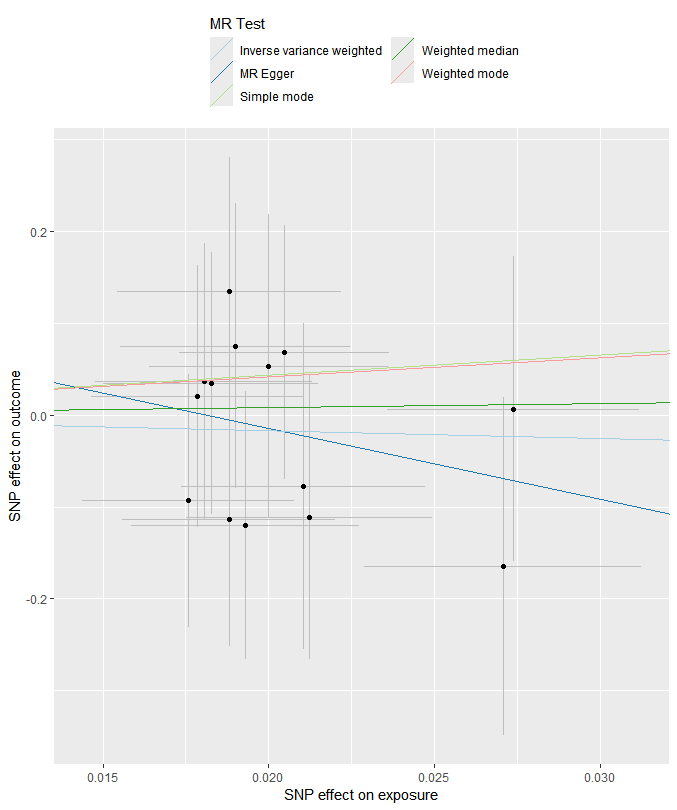


**Supplementary Fig. 5B: Leave-one-out sensitivity analysis for the association between mobile phone usage duration and testicular dysfunction. Each point represents the MR estimate obtained after omitting one SNP at a time. The stability of the estimates indicates that no single SNP has a disproportionate effect.**


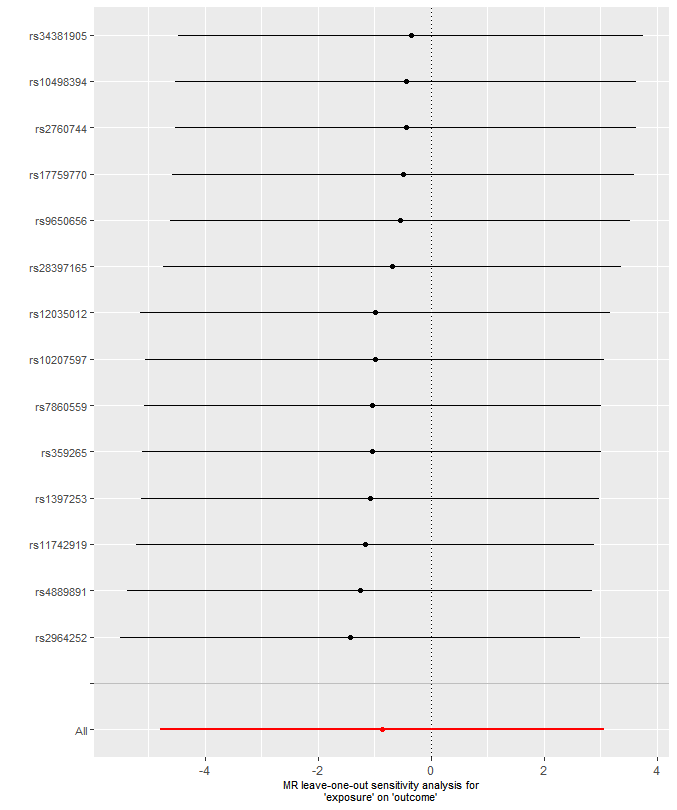


**Supplementary Fig. 5C: Forest plot showing the individual SNP estimates and the overall MR estimate for the association between mobile phone usage duration and testicular dysfunction using the IVW method.**


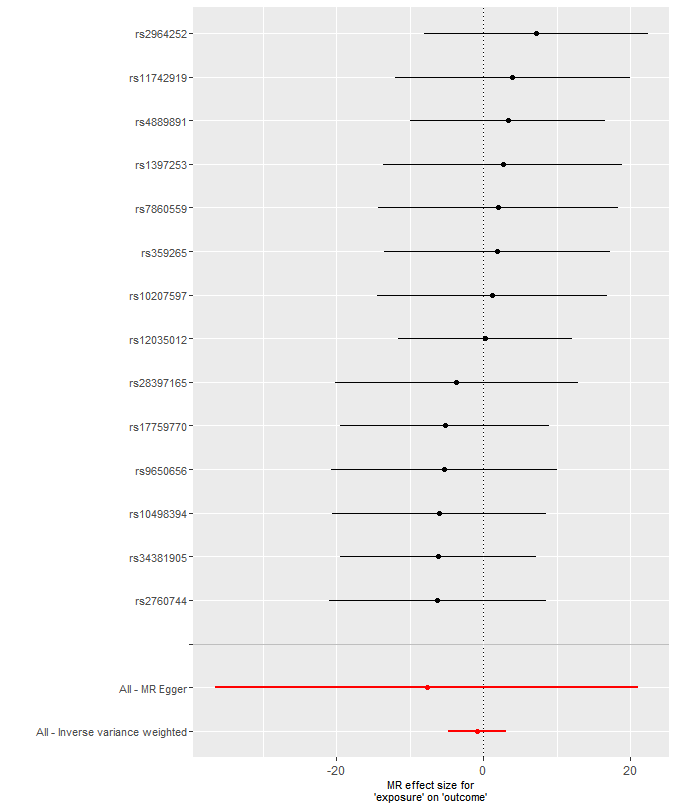


**Supplementary Fig. 5D: Funnel plot assessing potential small-study bias in the MR analysis of mobile phone usage duration and testicular dysfunction. The symmetry suggests no substantial directional pleiotropy.**


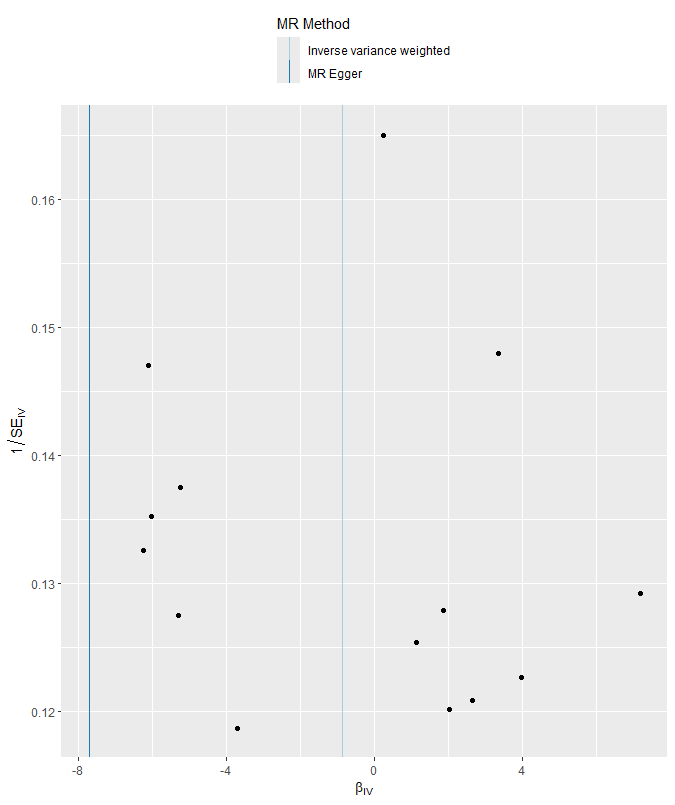

Supplement: Supplementary file 1 [file medi-104-e44668-s001.docx]
